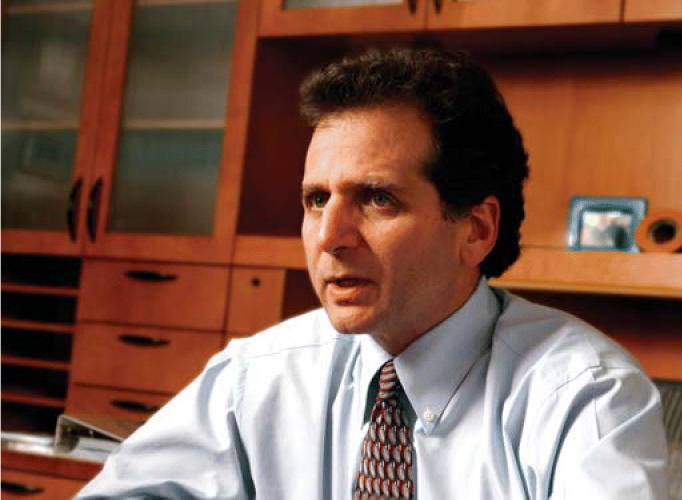# Considering a Society of Environmental Health Science

**DOI:** 10.1289/ehp.114-a142

**Published:** 2006-03

**Authors:** David A. Schwartz

**Affiliations:** Director, NIEHS and NTP, E-mail: david.schwartz@niehs.nih.gov

Although it may seem counterintuitive, the more we learn about science, the more complex it becomes. This is particularly true in the field of environmental health science. As the field has evolved, we’ve come to realize that the environment permeates nearly every question of human disease and that various forms of environmental stress can be used to understand many of the secrets of human biology. And the challenges that lie before us in solving the mysteries of these relationships will require the combined knowledge of a diverse array of scientific thinkers. For this and other reasons, the time is ripe for the field of environmental health science to consider the benefits of establishing a broad, vigorous, engaged scientific society.

One definition of society is “a structured community of people.” This also defines a predominant function of scientific societies: to provide a structured means for scientists to engage with other scientists. Never has this function been more important or necessary. The questions being asked by today’s environmental health scientists require multi- and cross-disciplinary approaches to answer, as well as scientific cooperation and interaction on a broader scale than ever before. Toxicologists must talk to epidemiologists. Geneticists must talk to ethicists. Biologists must talk to physicians. And governments, industries, academics, and advocates must talk to each other. A broad-reaching society for environmental health science could help to facilitate and promote such interdisciplinary interactions in both traditional and innovative ways. Traditional activities such as conferences, meetings, and membership subsections would allow for regularly scheduled discourse. Meanwhile, newer tools including websites, e-mail newsgroups, Internet conferencing, and weblogs could be used by a society to promote nearly instantaneous interaction among its members.

The time is ripe for the field of environmental health science to consider the benefits of establishing a broad, vigorous, engaged scientific society.

Indeed, helping to coordinate scientific discourse and the dissemination of scientific information has always been a major role of scientific societies. The need for this is even more apparent today. New technologies in fields such as bioinformatics and genomics, with which environmental health science is inextricably linked, are producing mountains of potentially valuable data. To compile, organize, mine, and disseminate such data for application to real health questions are enormous tasks. A society of environmental health science could be instrumental in coordinating these activities by facilitating their development, supporting their growth, and ensuring their dissemination through a variety of means including a credible peer-reviewed journal encompassing all aspects of the field.

It is not only today’s scientists that would benefit by membership in a vital and active environmental health science society. One of the greatest benefits of a scientific society to both individuals and entire disciplines is the training of future scientists; indeed, many societies engage in science education and training as an integral part of their mission. Now is a crucial period for the field of environmental health science in terms of the recruitment, training, and retention of promising young scientists, both here in the United States and especially in developing countries. A society of environmental health science could help in these efforts by developing, promoting, and providing innovative science education and training activities itself, as well as by helping to direct the training efforts of other institutions through consultation and advice. As an entity acutely in touch with the current reality of the field, a society would be in a unique position to help provide the types of scientists that will be needed to both focus and expand the field on its future trajectory.

Although a number of existing scientific societies address certain facets of environmental health science, no one society currently provides an encompassing and specifically focused source of support for the field. Some say this is because environmental health science has not been succinctly or easily defined in the past. Scientific societies exist, however, with a focus as broad as all of science and as narrow as a single biological function or genetic process. Furthermore, the purpose of a scientific society is not to define a field—that is done by the members themselves and by the contributions they make to science and society—but to support scientific endeavor to the benefit of its members and, ultimately, humankind. Rather than competing, a society dedicated to the support and furtherance of environmental health science would complement the mission of existing societies and, in fact, could enhance service to their memberships by partnering in common goals and activities. In return, current societies might be encouraged to broaden their mission to embrace aspects of the field of environmental health science.

What will be needed for the formation of such a society? Enthusiasm for the future direction of the field. Open minds willing to entertain new and diverse perspectives. Organization and a clear purpose of support for the field. And most important, leadership by active, engaged, committed scientists. The field of environmental health science is poised to break through to ever more amazing discoveries. A society of environmental health science could help such discoveries to come quicker and be more rapidly translated into prevention of disease and improvements in human health.

## Figures and Tables

**Figure:**